# The Role of Liposomal CpG ODN on the Course of *L. major* Infection in BALB/C Mice

**Published:** 2010-03

**Authors:** H Hejazi, M Tasbihi, Mr Jaafari, A Badiee, N Pestechian, A Javadi, A Khamesipour

**Affiliations:** 1 Department of Parasitology, Isfahan University of Medical Sciences, Isfahan, Iran; 2 Biotechnology Research Center and Nanotechnology Research Center, School of Pharmacy, Mashhad University of Medical Sciences, Mashhad, Iran; 3 Department of Social Medicine, Qazvin University of Medical Sciences, Qazvin, Iran; 4 Center for Research and Training in Skin Diseases and Leprosy, Tehran University of Medical Sciences, Tehran, Iran

**Keywords:** CpG ODN, Liposome, *Leishmania major*, BALB/c

## Abstract

**Background:**

Historically, leishmanization is the most effective protective measure against Cutaneous Leishmaniasis (CL), CL lesion induced by leishmanization sometimes takes a long time to heal. Manipulation of leishmanization inoculums needed to induce a mild and acceptable CL lesion. The aim of this study was to explore if liposomal form of CpG ODN (Cytosin phosphate Guanin Oligodeoxynucleotides) mixed with *Leishmania major* would induce a milder lesion size in Balb/c mice.

**Methods:**

This study was performed in Biotechnology Research Center, Mashhad, and Center for Research and Training in Skin Diseases and Leprosy, Tehran, Iran during 2008–2009. Different groups of BALB/c mice were subcutaneously (SC) inoculated with *L. major* mixed with liposomal form of CpG ODN, or *L. major* plus free CpG ODN, or *L. major* mixed with empty liposomes or *L. major* in PBS. The lesion onset and the size of lesion were recorded; the death rate was also monitored.

**Result:**

Footpad thickness was significantly (*P*<0.01) smaller, death rate was also significantly (*P*<0.05) lower in the mice received *L. major* mixed with liposomal CpG ODN or free CpG ODN than control groups received *L. major* in PBS or *L. major* plus liposomes, also mice which received *L. major* mixed with CpG ODN in soluble form showed a significantly (*P*<0.001) smaller lesion size than control groups.

**Conclusion:**

CpG ODN seems to be an appropriate immunopotentiator mixed with *Leishmania* stabilate in leishmanization.

## Introduction

Cutaneous Leishmaniasis (CL) is a major health problem in some endemic areas, including Iran (1). Search for an effective vaccine against leishmaniasis seems to be the sole control measure. Various forms of *Leishmania* antigens, such as; recombinant *Leishmania* antigens and live attenuated parasites, have been used to immunize against murine model of leishmaniasis (2–4). Induction of protection in animal model of leishmaniasis is possible, but today there is no vaccine available against any form of leishmaniasis, regardless of global attempts (5–7). Leishmanization showed to be the most effective tool against CL (5, 8) (9). It was stopped due to lack of standardization, and unexpected prolonged lesion at the site of leishmanization (10, 5). Bacterial DNA enhances innate and adaptive immune responses (11, 12). Stimulation of immune responses by bacterial DNA is due to the presence of unmethylated Cytosine-Guanine nucleotides motif in DNA sequences. Synthetic oligodeoxynucleotides containing unmethylated guanine cytosine motif (CpG ODN) mimic the stimulating effect of bacterial DNA (11, 12).

Using CpG ODN as an adjuvant mixed with *L. major* ribosomal proteins or promastigote antigens or recombinant *Leishmania* protein induced protection and even curative effect on *L. major* infection (13–15).

Liposomes are artificial closed vesicles composed of concentric lipid bilayers, separated by aqueous domains and utilized as delivery systems for drugs, peptides, proteins and DNA (16, 17). Encapsulation of CpG ODN into liposomes extends the duration of CpG ODN activity (18).

In this study, *L. major* promastigotes were co-inoculated with CpG ODN encapsulated in liposomes or in free form subcutaneously into susceptible BALB/c mice. The lesion size and the death rate were evaluated in immunized mice and compared with the control groups.

## Materials and Methods

In this experimental study, performed during 2008–2009, preparation of liposoms and encapsulation of CpG ODN into liposomes were done in Biotechnology Research Center, Mashhad, Mashhad, Iran and animal experiments were done in Center for Research and Training in Skin Diseases and Leprosy, Tehran University of Medical Sciences, Tehran, Iran.

### Animals

Forty female BALB/c mice aged 6–8 weeks old were purchased from Pasteur Institute (Tehran, Iran). The mice were maintained in animal house of Center for Research and Training in Skin Diseases and Leprosy and fed with tap water and standard laboratory diet. Animals were housed in a colony room with a 12-h-12-h light-dark cycle at 21°C with free access to water and food.

### Parasites

*Leishmania major* (MRHO/IR/75/ER) was grown in Novy-MacNeal-Nicole (NNN) medium, and for mass production, the promastigotes were subcultured in RPMI (Sigma, Germany) supplemented with 0.2 mM L-glutamine, 100 U/ml penicillin, 100 µg streptomycin and 15% fetal bovine serum. The promastigotes were harvested at stationary phase (stationary phase was estimated by daily enumeration of parasite number).

CpG ODN:

CpG ODN 1826 (Microsynth, Balgach, Switzerland), was a 20-mer (5'-TCC ATG ACG TTC CTG ACG TT-3') with a nuclease-resistant phosphorothioate backbone containing two CpG motifs (marked as bold) known to show an immunostimulatory effect on Th1 response in murine model (19).

### Encapsulated of CpG ODN in liposome

Liposomes containing CpG ODN were prepared by the dehydration-rehydration vesicle (DRV) method (20). The lipid phase consisting of 1,2-distearoyl-sn-glycero-3-phosphocholine (DSPC), 1,2 dioleoyl propyl 3 trimethylammonium bromide (DOTAB) and cholesterol dissolved in chloroform:methanol (2:1:1,v/v) in a round bottom flask. The solvent was removed by rotary evaporation resulting in deposition of a thin lipid film on the flask^’^s wall. The lipid film was freeze-dried overnight to ensure total removal of the solvent. The lipid film was then hydrated and dispersed in distilled water using vortex at 55°C. The resulting empty multilamellar vesicles (MLVs) were converted to 100 nm small unilamellar vesicles (SUVs) using the mini-extruder (Avastin, Canada). The CpG ODN were then added to empty SUVs liposomes, dried with freeze-drier overnight and rehydrated by distilled water. CpG ODN, which remained not encapsulated, was removed from encapsulated ones by centrifugation at 14,000×g for 15 min at 4°C.

Optical microscope (Olympus, Germany) was used to study the morphological features of liposomes. The particle size distribution and mean diameter of liposomes were determined by a particle size analyzer (Malvern, UK).

### Encapsulation efficiency of CpG ODN into liposomes

The efficiency of incorporation (% entrapment) of CpG ODN in liposome was determined using UV absorption at 260 nm. The analysis was performed on supernatants following PBS washes. The percentage of entrapment was calculated as described before (17). The concentration of CpG ODN in the liposomes was adjusted to 10 µg/50 µl after purification and calculation of percent of entrapment.

### Induction of lesion in BALB/c mice

BALB/c mice (10 per group) were inoculated SC in the right footpad with 2×10^6^ *L. major*, promastigotes harvested at stationary phase mixed with either CpG ODN (10µg), liposomal CpG ODN (10µg CpG ODN), empty liposome or PBS in final volume of 60µl. The development of lesion was monitored and recorded in each mouse by weekly measurement of footpad thickness using a metric caliper. Grading of lesion size was done by subtracting the thickness of the uninfected contralateral footpad from that of the infected one (18).

### Statistical analysis

Repeated measurement ANOVA statistical test was used to assess the significance of the differences among various groups. Bonfroni test was used to compare the means of different treatment groups. Results with *p* values of<0.05 were considered statistically significant.

### Ethical considerations

Animal experiments were carried out according to Tehran University of Medical Sciences, Ethical Committee Acts and were approved by the TUMS Ethical Committee.

## Results

### Liposome characterization

The liposomes were morphologically multilamellar vesicles, as observed under optical microscope. The mean diameters calculated by particle size analyzer were 1.01±0.45 and 1.3±0.3 µm (n=3) for liposomal CpG ODN and empty liposome, respectively. The entrapment of CpG ODN in liposomes was estimated to be more than 95%.

### Lesion size

**Fig. 1:**
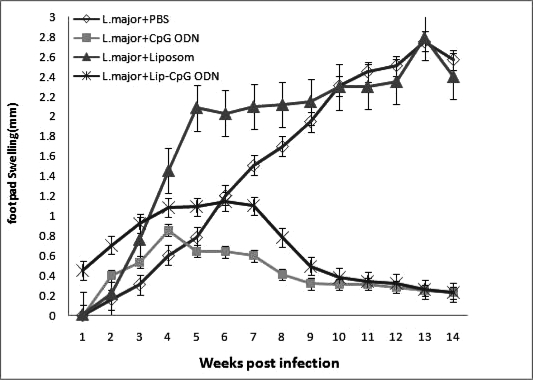
Footpad swelling in BALB/c mice inoculated in the right footpad with 2×10^6^ *L. major* promastigotes SC together with PBS, soluble CpG ODN, liposomal CpG ODN or empty liposomes. The footpad thicknesses of mice were measured for 14 weeks. Each point represents the average increase in footpad thickness±standard error of the mean (n=10)

Footpad swelling in mice received *L. major* mixed with liposomal form of CpG ODN was significantly (*P*<0.01) smaller than control groups which received *L. major* in PBS or *L. major* mixed with empty liposomes. In the group of mice, received *L. major* mixed with liposomal form of CpG ODN, only induration was induced and no ulcer was seen in the footpad of any of the mice. The mean average of footpad swelling in this group showed to increase up to 6 weeks after inoculation with maximum thickness of 1.14 mm and then the lesion size started to decreased thereafter which ultimately reached to 0.23 mm at week 14 (Fig. 1).

The average footpad thickness in the group of mice that received *L. major* mixed with free CpG ODN was significantly (*P*<0.001) smaller than groups received either *L. major* in PBS or mixed with empty liposomes. In the group of mice, which received *L. major* mixed with free CpG ODN, the average of footpad thickness increased up to 4 weeks after inoculation with maximum thickness of 0.85 mm and thereafter the lesion size started to decrease and reached to 0.20 mm at week 14 post-infection. In this group, only one out of 10 mice developed an ulcer on week 5, which was self-healing and completely healed by week 12.

Ulcer was seen in group of mice received *L. major* in PBS at week 5 post-inoculation with an average lesion thickness of 2.75 mm and from week 14, the infected foot started to fall.

The average thickness of lesion was 2.78 mm in mice received *L. major* mixed with liposomes. There was no significant difference in footpad swelling in mice received *L. major* with empty liposome and the mice received *L. major* in PBS.

There was no significant difference between the average of footpad swelling in the group, which received *L. major* in CpG ODN, and group, which received *L. major* in liposomal form of CpG ODN (Fig. 1).

### Death rate

In the group of mice, received *L. major* mixed with CpG ODN, only one (10%) mouse died at week 20 after infection and no more death was seen up to 6 months after the inoculation.

In group of mice received *L. major* mixed with liposomal form of CpG ODN, one mouse died at week 15, two mice died at week 18, one mouse died at week 19 and one mouse died at week 23 post-infection therefore a total of 5 mice (50%) died up to 6 months.

The mice that received *L. major* in PBS, 2 mice died at week 14, 3 mice died at week 15, one mouse died at week 16 and 4 mice died at week 17 post-infection, a total of 10 (100%) died up to week 17.

**Fig. 2:**
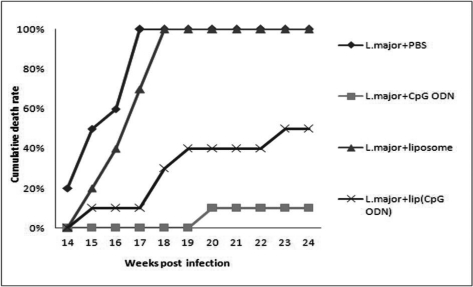
Death rate in BALB/c mice inoculated in the right footpad with 2×10^6^ *L. major* promastigotes SC together with PBS, soluble CpG ODN, liposomal CpG ODN or empty liposomes

Concerning the mice that received *L. major* with empty liposome, 2 mice died at week 15, 2 mice died at week 16, 3 mice died at week 17 and 3 mice at week 18 post-infection and a total of 10 (100%) died by week 18 (Fig. 2).

## Discussion

Leishmanization is considered the most effective tool to protect against CL. It is also an effective tool to evaluate efficacy of *Leishmania* candidate vaccines (5, 8–10). However, due to the problems associated with leishmanization such as lack of control over the lesion development and healing process, it was stopped except in Uzbekistan (5). CpG ODN as an immunostimulatory adjuvant was used as monotherapy against cancer (21), and in conjunction with an allergen to improve the immunogenicity of an antigen and at the same time to reduce its allergenicity (22). CpG ODN is also used to induce protection against infectious disease such as hepatitis B (23), listeriosis (24) and malaria (25).CpG ODN was used as an adjuvant mixed with different *Leishmania* antigens to induce protection in murine model of leishmaniasis (13, 14). The administration of *L. major* ribosomal proteins (LRP) along with CpG ODN induced protection against *L. major* infection in BALB/c mice. Footpad swelling of LRP+CpG-ODN immunized mice was significantly lower than the control group received LRP alone (14). In addition, immunization of BALB/c mice with autoclaved *L. major* (ALM) along with CpG ODN induced protection against challenged with *L. major* at week 12 after immunization (13). CpG ODN used in this study promotes Th1 response, the type of response required to induce protection in leishmaniasis (19). Mendez *et al.* (26) showed that when C57BL/6 mice infected intradermally with *L. major* mixed with 50µg CpG ODN with or without ALM showed a much milder lesions compared to the control groups, in addition the experimental group of mice were protected against *L. major* challenge for up to 6 months. BALB/c mice are highly susceptible to *L. major* infection, upon infection the mice develop skin lesions, which expand and metastasize, and eventually every mouse succumbed to the disease (27). In the present study, susceptible BALB/c mice were infected with *L. major* mixed with 10 µg of CpG ODN encapsulated in liposomes or in free form, the lesion development and death rate was compared with the control groups.

The results showed that co-administration of CpG ODN with *L. major* in susceptible BALB/c mice even with a lower dose i.e. 10 µg compared with 50 µg used previously by others (26), induced a significantly smaller lesion size with significantly lower death rate. Liposomes as system for carrying drugs, proteins, and DNA protect encapsulated content from damages caused by environmental enzymes like endonuclease (16). In a study, BALB/c mice immunized with rgp63 plus CpG ODN encapsulated in liposomes or rgp63 mixed with CpG ODN in soluble form, upon challenged with *L. major,* there was a significant (p<0.05) difference between the lesion size in mice immunized with rgp63-CpG ODN and the control group up to week 11 after challenges but thereafter the lesion size was not significantly different, but the lesion size in group which received rgp63-lip-CpG ODN up to 14 weeks post challenge was significantly (*P*<0.001) smaller than the control group (18).

In the present study, the group of mice received *L. major* mixed with CpG ODN encapsulated in liposome, only indurations were seen in the experimental group of mice and no ulcer was seen up to 6 months after infection. No significant different was seen between averages of footpad swelling of the group which received *L. major* mixed with CpG ODN encapsulated in liposomes, and the group received *L. major* mixed with free form of CpG ODN.

The groups of mice that received *L. major* mixed with CpG ODN encapsulated in liposomes, or *L. major* mixed with free form of CpG ODN, a significantly (*P*<0.05) lower death rate was seen compared with the control groups up to 6 month period after inoculation; one death out of 10 mice in group which received *L. major* mixed with CpG ODN in free form and 5 death out of 10 mice in group received *L. major* mixed with CpG ODN encapsulated in liposome were seen. Although the death rate was lower in group of mice received *L. major* mixed with CpG ODN in free form than the group which received *L. major* mixed with CpG ODN encapsulated in liposome but there was no significant difference in lesion size between the two immunized groups.

To increase immunostimulatory, CpG ODN was encapsulated into liposomes, phosphorothioate CpG ODN which is used in this study is resistant to *in vivo* degradation by endonuclease enzymes. Based on this theory, it was anticipated that encapsulation of CpG ODN in liposomes will be resulted in a lower lesion size and a lower death rate in mice. However, as shown in Fig. 1, there is no significant difference between mice received CpG ODN in liposomal form and mice received CpG ODN in soluble form, which might be due to the lipid used in liposome formulation (i.e. DSPC). DSPC has a very high transition temperature (Tm 55°C) and produces a very rigid and stable bilayer structure in liposome formulation (28) and as a result, it does not destroyed easily in phagosome of target cells. Hence, there would not be enough free CpG ODN available in phagosome to interact with its specific receptor, i.e. TLR9, localized in phagosome (29). In terms of mice, which received free form of CpG ODN, there is one theory that free CpG ODN may interact with TLR9 receptor more effectively than those encapsulated in very rigid and stable liposomes, also phosphorothioate backbone in this type of CpG ODN is not damaged by endonuclease *in vivo*. The results of current study suggest that further studies are needed to identify a suitable lipid to formulate the liposomes, which release their content, i.e. CpG ODN, on an appropriate time to interact with TLR9.

In conclusion, the results showed that co-inoculation of CpG ODN with *L. major* induce a milder leishmanization lesion compared to the control group and might be appropriate to use in combination with it.
